# Quantitative measures of healthy aging and biological age

**DOI:** 10.12715/har.2015.4.26

**Published:** 2015-04-23

**Authors:** Sangkyu Kim, S. Michal Jazwinski

**Affiliations:** 1 Tulane Center for Aging and Department of Medicine, Tulane University Health Sciences Center, New Orleans, LA, USA

## Abstract

Numerous genetic and non-genetic factors contribute to aging. To facilitate the study of these factors, various descriptors of biological aging, including ‘successful aging’ and ‘frailty’, have been put forth as integrative functional measures of aging. A separate but related quantitative approach is the ‘frailty index’, which has been operationalized and frequently used. Various frailty indices have been constructed. Although based on different numbers and types of health variables, frailty indices possess several common properties that make them useful across different studies. We have been using a frailty index termed FI_34_ based on 34 health variables. Like other frailty indices, FI_34_ increases non-linearly with advancing age and is a better indicator of biological aging than chronological age. FI_34_ has a substantial genetic basis. Using FI_34_, we found elevated levels of resting metabolic rate linked to declining health in nonagenarians. Using FI_34_ as a quantitative phenotype, we have also found a genomic region on chromosome 12 that is associated with healthy aging and longevity.

## Introduction

The importance of health span as opposed to life span has gained substantial recognition over the past decade. Health span is defined as the period of life spent in relative good health. This definition carries with it the necessity to quantify ‘healthy’ versus ‘unhealthy’ aging, in order to understand the variables contributing to health span. The problem of how to quantify health span has occupied researchers for some three decades, and it has both basic scientific as well as applied clinical ramifications.

Much work in the field of the biology of aging has focused on individual cellular and molecular mechanisms as causal factors restricting longevity. This has led to a wealth of information that has gained particular predictive value with the introduction of genetics, especially in lower organisms. However, there has always been an appreciation for aging as a manifestation of the organism as a whole, which immediately calls attention to integrated function and its decline in the form of physiologic dysregulation. Thus, the search for descriptors of this whole-organism functional decline has resulted in the elaboration of various indices of healthy versus unhealthy aging. This search has taken into account the heterogeneity of the aging phenotype from individual to individual over space and time; a remarkable feature of aging common to a number of species [1]. The tendency to view healthy aging in a holistic sense is fundamentally a systems biology perspective on aging and health [2].

The anecdotal finding of reduced disease burden in long-lived individuals has been frequently mentioned in the scientific literature, and has been underpinned by the quantitative classification of centenarians as survivors, delayers, or escapers of major diseases [3]. However, careful analysis has shown that there is no difference between centenarians and young controls in the frequencies of genetic variants predisposing individuals to major diseases of aging [4]. Nevertheless, it has been shown recently that individuals from families enriched for persons displaying exceptional survival exhibited a marked delay in the onset of age-related diseases and comorbidities [5], suggesting a genetic component. Indeed, such genetic factors have been identified [6]. Diseases and disorders of aging have figured into other measures of healthy aging, but in and of itself, absence of disease is not useful when categorizing healthy aging, since few people escape unscathed with increasing age.

The concept of ‘successful aging’ [7] is an attempt to quantify health span as opposed to life span. Successful aging is defined as having a low level of disease and/or disease-related disability, relatively high physical and cognitive functioning, and active and productive engagement in life activities. This construct has been operationalized and used directly in genetic studies of aging [8].

Frailty is considered a clinical syndrome that distinguishes elderly individuals at risk for adverse outcomes. It does so by quantifying the functional loss that results during aging [9, 10]. This has led to several frailty indices. Frailty was defined by Fried *et al*. [11] based on the presence of at least three of a possible total of five deficits: weight loss, exhaustion, muscle weakness, slow walking speed, and low physical activity. As expected, the prevalence of frailty increases with age. Studies designed to uncover genes that play a role in frailty have been based on assumptions about the underlying mechanisms; i.e., the secondary phenotypes or endophenotypes involved [12, 13].

The clinical syndrome of frailty as defined above is most appropriately considered a phenotype. It is considered distinct from disability, which is often measured in the elderly as impairment in the performance of activities of daily living (ADL). It is also distinguished from comorbidity. There is some overlap between the three conditions across a cohort of older individuals [11]. The major difference between the frailty phenotype and disability or comorbidity is that with frailty, there is the assumption of decreased functional reserve and physiologic dysregulation that results in a reduced ability to recover from destabilizing stress. This suggests that the frailty phenotype is useful for uncovering underlying biological mechanisms. It is also predictive of disability [14], which may allow its use in understanding the factors determining individual trajectories of disability [15].

A somewhat different approach to quantifying frailty involves a frailty index (FI), consisting of the fraction of deficits accumulated by an individual out of a total of 92 health variables [16]. These variables encompass a broad array of indicators of decline in various physiologic systems throughout the body, and they group together symptoms, laboratory measurements, diseases and disabilities. FI is a better predictor of longevity than chronologic age – in essence, it is a measure of biologic age. Subsequently, it was determined that far fewer variables need be included to achieve an informative index, as long as they reflected the function of a spectrum of physiologic systems [17, 18]. In some studies, the term ‘deficit index’, rather than frailty index has been used [19]. One feature that can complicate use of the FI is its inclusion of disability and comorbidity among its variables. However, their use in the index can be constrained when the relationship of frailty to disability and comorbidity is examined. Claims that use of FI to describe frailty make investigation of underlying mechanisms impossible are unwarranted, as will be seen below.

Recently, a hybrid approach to frailty was applied to two distinct geographic populations [20]. This clustering approach incorporates select features of successful aging, frailty phenotype, and FI. It successfully classifies individuals into different frailty groups differing by mortality risk. It displays a narrow sense (additive) heritability of 0.43 – this compares favorably with the heritability of longevity, which ranges from 0.15 to 0.35 in different estimates [21, 22]. However, the genetic contribution to longevity increases with age [23].

A concept that developed concurrently with frailty is ‘allostatic load’ [24], which attempts to characterize the effect of cumulative biological burden as the body adapts to life stress. When this load exceeds a hypothetical threshold, the resulting wear and tear compromises the physiologic regulatory systems, leading to failure to adapt. Allostatic load has a strong biologic rationale, and it incorporates assessments of ten biomarkers that reflect the operation of several regulatory systems and processes. Baseline allostatic load predicts longitudinal mortality, as well as changes in physical and cognitive functioning.

Another approach utilizing biomarkers attempts to quantify the physiologic dysregulation that is at the root of frailty. These biomarkers were selected in two separate groupings [25]. The ‘statistical suite’ of biomarkers was selected on the basis of the significant increase with age of the deviation of the biomarker from the population average value at baseline. The ‘biological suite’ consisted of those biomarkers most strongly associated with the first axis of variation in a principle component analysis that was stable across three different populations. Individuals were classified by the multivariate statistical difference of their deviation (D_M_) from the centroid of a reference population characterized by healthy physiology. It was shown that D_M_ accelerates with age, and is associated with increased risk of various health outcomes including mortality and frailty, after adjusting for age. The effort to uncover biomarkers of aging has also encompassed the epigenetic level in the form of DNA methylation marks of human cells and tissues [26].

A related multivariate approach to those listed above utilized principal component analysis to identify endophenotypes of a long and healthy life [27]. The individual variables incorporated into the analysis included an array of measures of physical and cognitive function, as well as physical examination and laboratory measures. The most dominant principal component accounted for 14.3% of the variability across the sample, and was composed of measures of physical function, metabolic health, and pulmonary function. It had a narrow sense heritability of 0.39. Interestingly, average and maximum handgrip strength, and HDL cholesterol levels, which were included in this principal component, had somewhat higher heritability. The importance of physical function ability in predicting survival is well known [28], thus the inclusion of physical function in this principal component is not surprising.

In this article, we describe the derivation and properties of an FI we are using in our analyses of genetic and phenotypic aspects of healthy aging. We highlight its performance juxtaposed to the performance of various other measures of healthy aging, in cases in which this is possible due to the availability of relevant comparable information.

## The frailty index

The semi-quantitative approach to frailty based on a small number of items may allow relatively quick screening of frail people and affected body domains [17, 29]. However, it is not considered to be comprehensive or sufficiently quantitative, rendering it less useful in assessing healthy aging at the whole organism level [30]. The FI introduced by Mitnitski *et al*., which is based on a set of 92 health variables, includes many different health variables reflecting different types of body systems [16]. It was intended to compile a broad spectrum of age-related changes that occur in multiple biological domains. Thus, rather than focusing on single markers of aging that may vary widely and give biased characterization of aging, this FI aims to characterize aging in an integrative and systemic way for the whole organism. Since then, various FIs or deficit indices with different numbers and types of health variables have been used and studied [17, 18, 31–33].

An individual’s FI score is the proportion of any deficient health variables in a set of health variables surveyed for the individual at a given age. Collected data for health variables are usually quantitative measures, either continuous or discrete, or categorical responses from medical history questionnaires. Binary categorical responses are numerically coded; 0 for the absence of the deficit and 1 for the presence of the deficit. Quantitative data and multi-categorical responses are re-coded in the same way as reported previously [33, 34], or with appropriate modifications as shown in Table 1.

Thus, FI scores range from 0, which means no deficient variable in all the health variables surveyed, to 1, which means deficiency in all the health variables surveyed. Accordingly, we constructed an FI based on 34 health variables (FI_34_) and studied its properties as a composite phenotype of healthy aging [35]. Our 34 variables include diseases and symptoms throughout the body, deficiencies in physical and cognitive functioning, and self-rated health status (Table 1). We have been using FI_34_ in genetic and phenotypic analyses of healthy aging.

## Properties of FI_34_ and other frailty indices

Most of the data on FIs are from cross-sectional studies; hence the exact age trajectory of some of their properties may differ over time. Nevertheless, some interesting statistical and demographic properties have emerged from comparisons of different FIs available in the literature. The foremost features, which make the FI extremely useful across different studies, is that it is robust and consistent from study to study, as long as the number of health variables is statistically valid and sufficiently diverse to represent multiple body domains [16, 18].

### Distribution of FI scores

**Figure 1:**
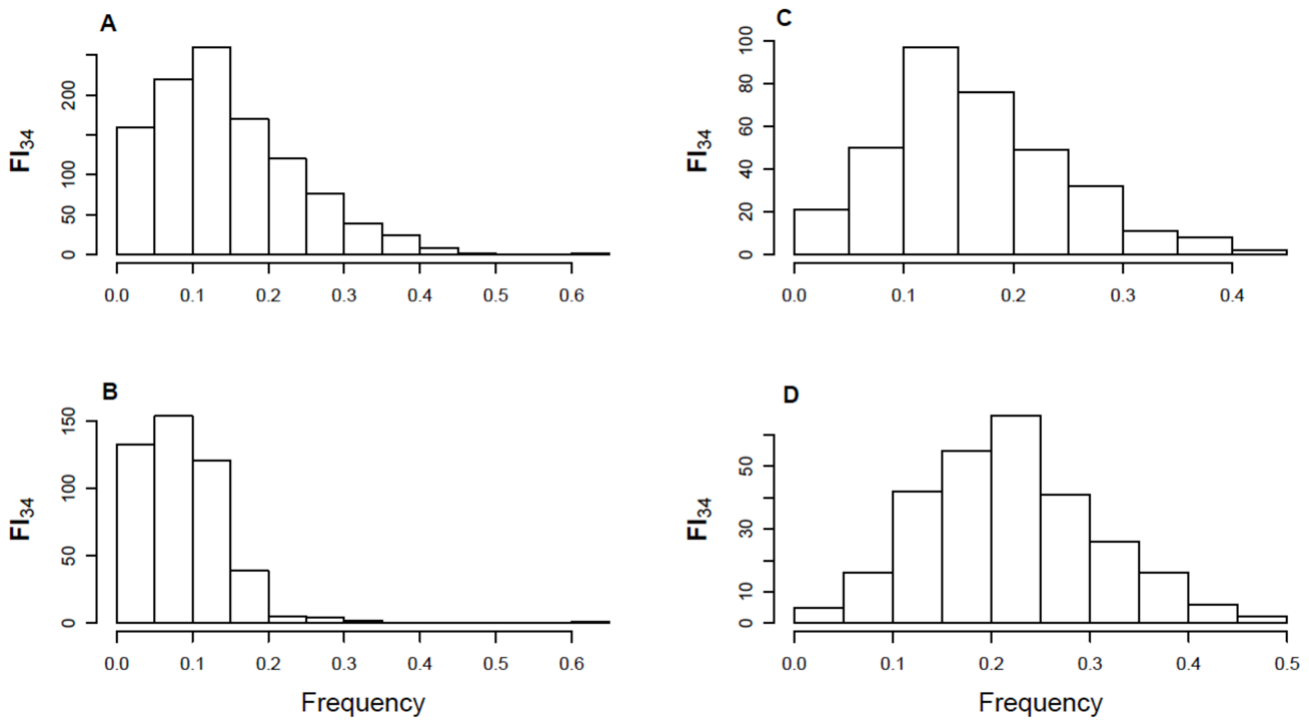
Distribution of FI_34_ scores of individuals in the Louisiana Healthy Aging Study (LHAS) and the Healthy Aging Family Study (HAFS). The FI_34_ scores were compiled for subjects in LHAS [76] and HAFS [35], according to the methods described [35]. Shown are all the age groups (A), 459 young individuals (20–60 years old) (B); 348 middle-aged (60–90 years old) (C), and 382 old (90–104 years old) (D).

The distribution of FI scores is usually positively skewed (Figure 1A), which is best fit by the gamma density function where two parameters determining shape and scale are involved [16]. Demographically, the distribution of FI scores changes depending on the age groups considered (Figure 1B–D). Since the FI is highly correlated with age, the skewed distribution reflects the presence of healthy groups (gamma distribution) and unhealthy frail groups (normal distribution). Longitudinally, the two-parameter distribution might represent two-stage changes, where the first stage corresponds to individuals’ resilience to the deleterious changes, and the second stage to the deteriorating stage of declining function with age [16].

### Non-linear increase in the rate of deficit accumulation

**Figure 2:**
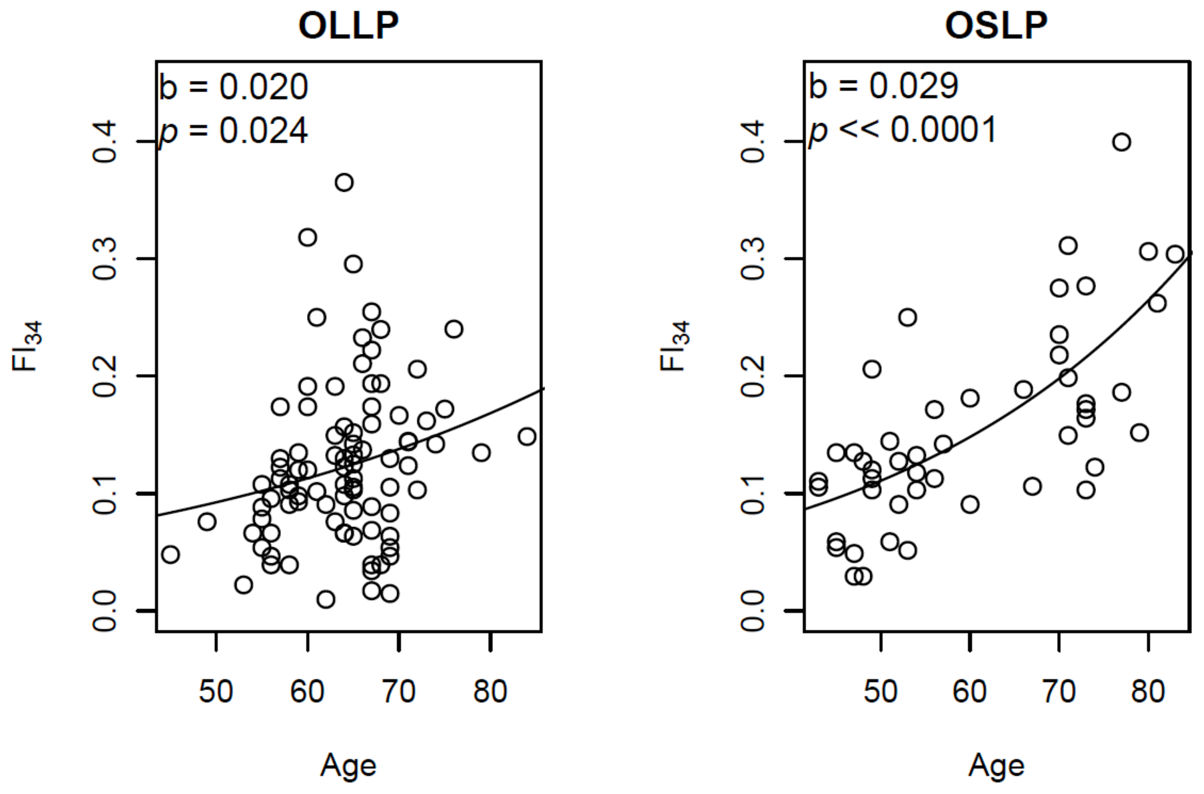
Scatter plots of FI_34_ scores by age in the “offspring of long-lived parents” (OLLP) of the Healthy Aging Family Study and the “offspring of short-lived parents” (OSLP) of the Louisiana Healthy Aging Study. Using the FI_34_ as a dependent variable and age as an independent variable, the exponential function a•e^(b•age)^ was fitted to estimate the parameters a and b. The value of a=0.034 for OLLP and 0.026 for OSLP. Shown are the estimated b values with corresponding *p* values under the null hypothesis that slope =0. Reproduced with permission from [35] with modifications.

**Figure 3:**
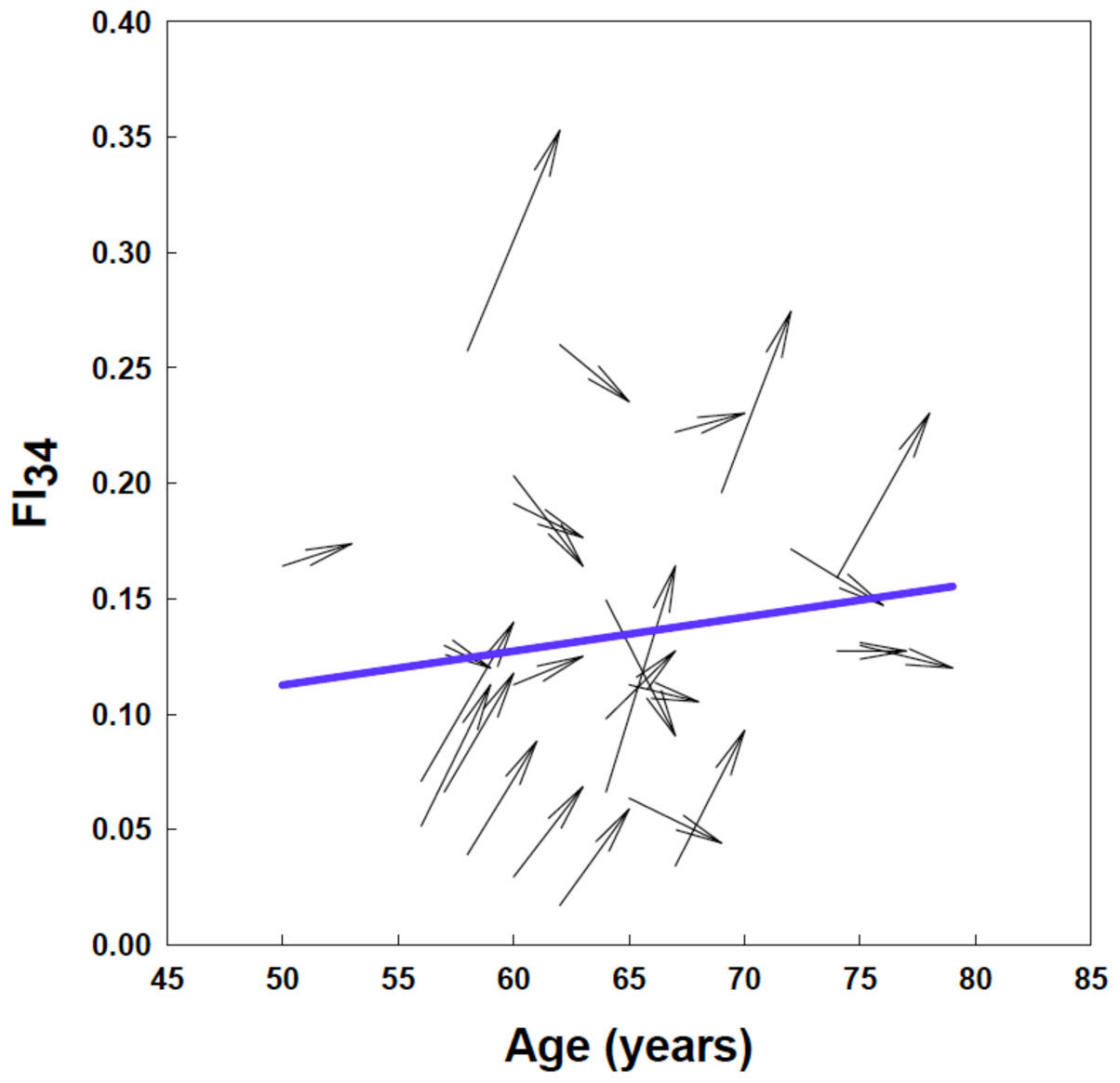
Age trajectories of FI_34_ scores of individuals in the Healthy Aging Family Study [35]. FI_34_ scores can decline individually as noted previously [38], but the population or group statistic of FI_34_ increases over time. The plots (arrows) are from two data sets collected over a three- to four-year interval from 25 HAFS participants who were 50 to 75 years old at the time of collection of the initial data set. The blue line is the average FI_34_ for this group of subjects.

FI is highly correlated with age and increases nonlinearly with increasing age. The non-linear relationship is best fit either by an exponential function or by a quadratic equation [16, 36]. Interestingly the rates of accumulation of deficits with age calculated from different numbers of health variables (e.g., from 20 to 92) are all close to ~2–3% per year. With FI_34_, the instantaneous rate of deficit accumulation falls within this range (Figure 2). The seemingly narrow range of rates may reflect insensitivity of the FI to the choice of particular items. This robustness may also come from the redundancy of variables, which may further reflect interrelationships of different body systems. Thus, redundancy is a statistical phenomenon, but it may well be based on functional relatedness between variables. It is important to remember that this continuous increase in FI_34_ is a population phenomenon. We have found that FI_34_ can increase, decrease, or remain unchanged over a period of three to five years (Figure 3).

The non-linear increase in FI with age may represent increased vulnerability to stressors as health deteriorates [37]. Indeed, the chance of having higher numbers of deficits increases as the number of deficits accumulated increases [38]. This acceleration is an example of a feed-forward mechanism, and is characteristic of the operation of a complex system in which there are multiple interactions among its individual components. Interestingly, however, no differences in the rate of deficit accumulation were observed between ‘healthy’ individuals who did not contract any of 21 major diseases and ‘unhealthy’ individuals who contracted at least one of these [36]. In this case, the numbers of health deficits at baseline were higher in the unhealthy than in the healthy individuals. If contracting one or more of the major diseases is associated with frailty, then the rate of deficit accumulation in the unhealthy should be higher than that in the healthy.

One possible reason for this discrepancy could be that frailty is fundamentally different from the occurrence of diseases. In other words, frailty progression with advancing age is little affected by the presence of diseases. Disability may be more significant than comorbidity in assessing frailty because disability can alter the aging pattern [36] and frailty leads to disability [29, 39]. In fact, disability was not taken into consideration when the subjects were categorized into ‘healthy’ and ‘unhealthy’ according to disease histories (i.e., ‘healthy’ individuals could have disability) [36].

Mitnitski *et al*. [40, 41] used a mathematical model to explain the non-linear accumulation of deficits: the average number of deficits present in an individual is the product of the average intensity of the environmental stresses and the average recovery time. According to this model, an individual’s frailty is the outcome of two competing factors: environmental damage and the ability to cope with that damage. Environmental damage is regarded as a stochastic process, but the ability to recover from damage depends on an individual’s assets, such as genetic endowment, health status, living conditions, access to health care, etc. This is a simple but useful model; not only does it account for the exponential increase in the rate of deficit accumulation, but also it emphasizes the importance of environmental factors in healthy aging.

### Gender specificity

Some FI properties may be gender-specific. For example, women accumulate more deficits than men of the same age, but their risk of mortality is lower [42]. This observation is in line with the result of a separate study showing that within an age group, females have overall worse health than males even though they live longer [43]. In a different cross-sectional study however, no gender differences were observed in the rate of deficit accumulation [36]. The discrepancy may be due to the presence or absence of gender-specific health dimensions in the health variables used to calculate FI score [36]. Our FI_34_ does not include any explicit gender-specific health variables (e.g., prostate-related pathologies) and does not show any significant gender differences in various analyses [35].

### An indicator of biological aging

FI is a reliable indicator of biological age and predictor of survival/mortality [16, 31, 42, 44, 45]. As the number of deficits accumulated increases, the risk of mortality increases exponentially [38]. Where individuals are of the same age but have different FI scores, the individual with the higher FI score is more likely to die sooner. Chronological age may even be ignored if FI is used to predict adverse outcomes. We tested FI_34_ for its predictive role in survival/mortality (Table 2). As expected, both age and FI_34_ were significantly associated with survival times, which included both censored and uncensored data (*p*<0.0001 for both). However, when the Cox proportional hazard regression was limited to time to death (uncensored survival times), only FI_34_ had a significant effect on the hazard of death, whereas chronological age did not (*p*=0.0048 for FI_34_ vs *p*=0.12 for age). These results indicate that the FI_34_ performs as well as other FIs.

### A tool to identify physiologic factors associated with healthy aging

**Figure 4:**
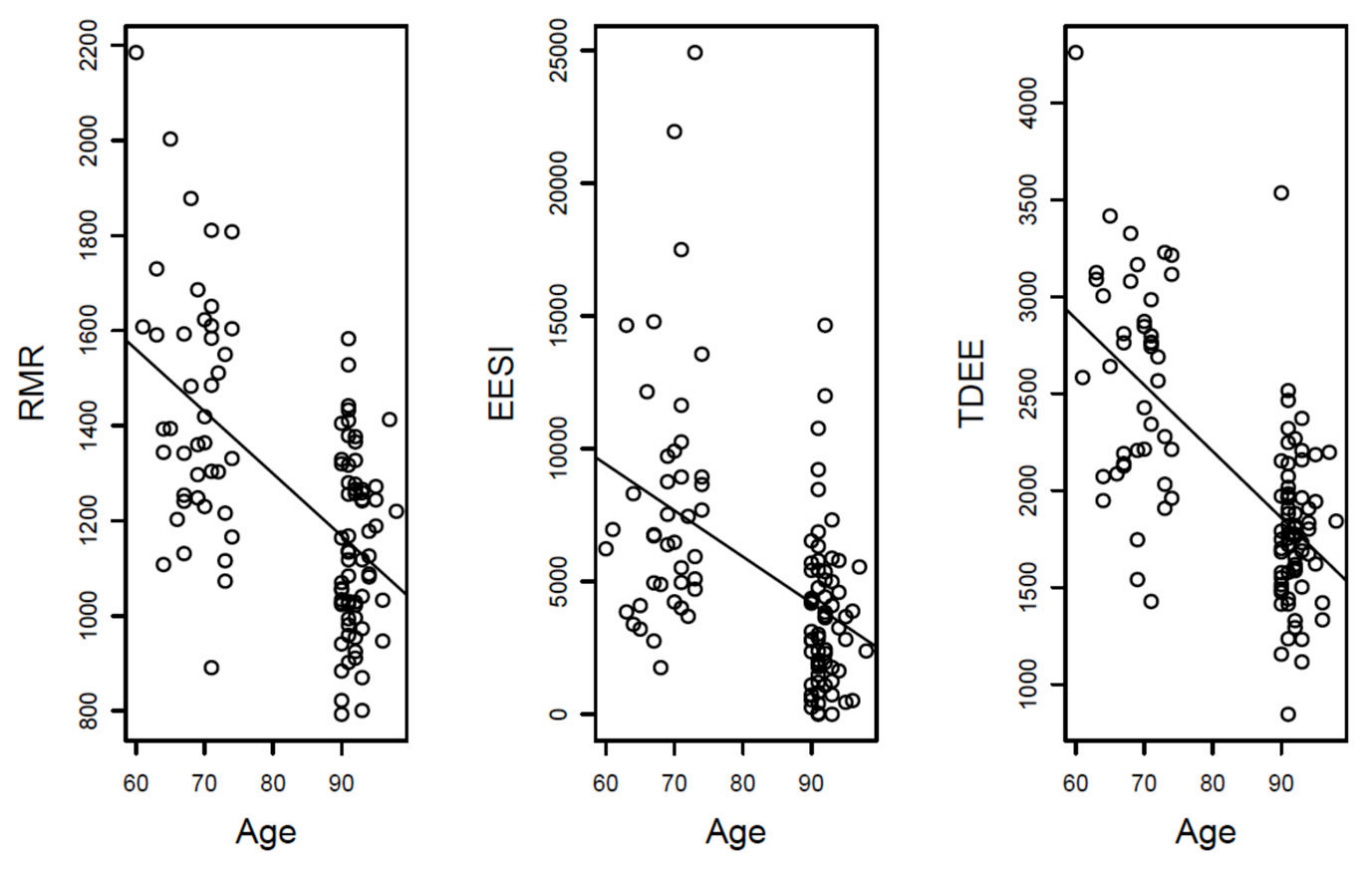
Energy expenditure components are inversely correlated with age in the Louisiana Healthy Aging Study. Energy expenditure associated with physical activity is represented by the energy expenditure summary index (EESI) in the Yale Physical Activity survey. The plots were generated using data from 109 study participants aged 80–98. RMR, resting metabolic rate; TDEE, total daily energy expenditure.

As a quantitative proxy of aging and longevity, the FI can be used to examine various physiologic or genetic factors for their contribution to healthy aging. To do so, we turned our attention to energy metabolism, which is indispensable to life [46]. Total daily energy expenditure (TDEE) in mammals can be divided into three major components: resting metabolic rate (RMR), activity energy expenditure (AEE), and diet-induced thermogenesis [47–49]. RMR, which accounts for the bulk (60–70%) of TDEE, refers to the amount of energy for maintenance of body systems [49]. AEE and diet-induced thermogenesis constitute approximately 20–30% and 10% of TDEE, respectively. These essential components of energy metabolism are highly associated with age (Figure 4), and in examining their relationship with FI_34_, we included several covariates known to be related to the independent or dependent variables. These variables include age, gender, fat mass, fat-free mass, the thyroid hormones T3 and T4, insulin-like growth factor 1 (IGF1), and creatine phosphokinase (CPK). Of these, IGF1 has the potential to affect RMR by inducing skeletal muscle growth through activation of the Akt-mTOR pathway [50, 51]. CPK is a clinical indicator of muscle damage [52–54].

First, we noticed that RMR is positively associated with FI_34_ among nonagenarians, as shown in Table 3 (gender-adjusted regression coefficient=1.60·10^−4^, *p*=3.4·10^−3^). In other words, RMR increases as FI_34_ increases. This finding was somewhat unexpected because of the inverse correlation of RMR with age (Figure 4): we expected lower RMR in older individuals. However, this is the first time that this association has been examined as a function of healthy versus unhealthy aging in the oldest-old. We also found that CPK is positively associated with FI_34_, but only in males (Table 3). On the other hand, fat-free mass is inversely correlated with FI_34_ in females only. We regard the increase in RMR as a mechanism to maintain homeodynamics as health declines in the oldest-old. The results also indicate that details of the association of energy expenditure with healthy aging may be different between the two genders.

**Figure 5:**
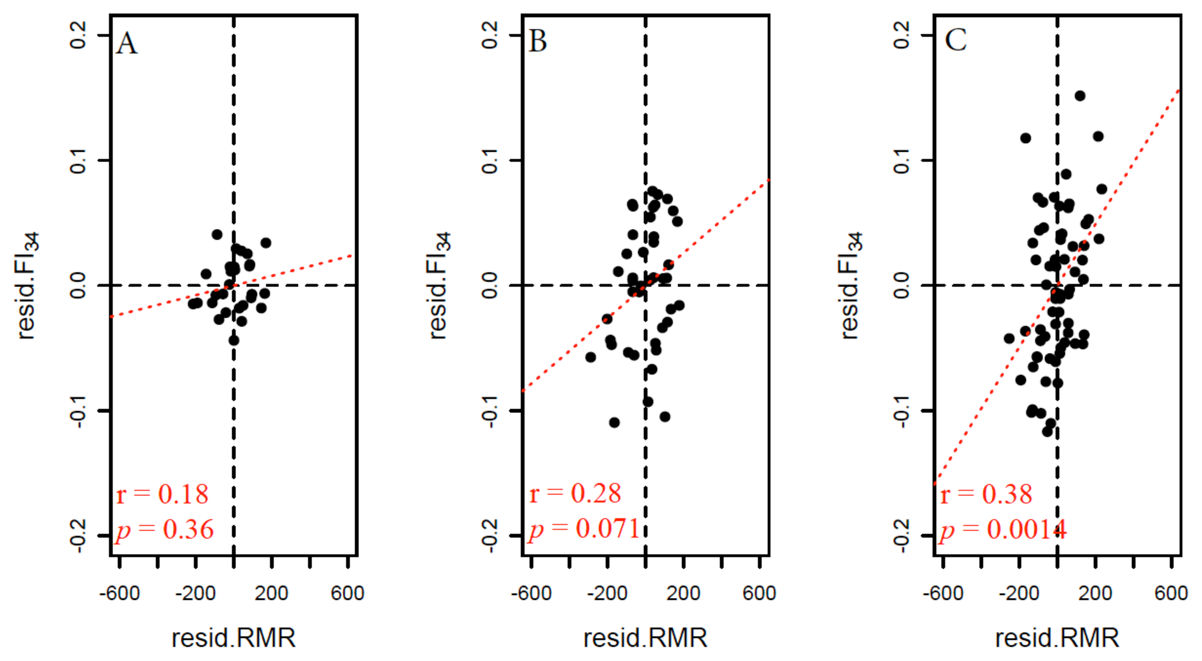
Age-dependent variation of FI_34_ and RMR. The “resid.FI_34_” on the y axis represents residuals (the differences between the observed FI_34_ scores and the predicted FI_34_ scores) from a linear regression of FI_34_ on age with adjustments for sex, fat mass and fat-free mass. Likewise, “resid.RMR” on the x axis represents residuals (the differences between the observed RMR scores and the predicted RMR scores) from a linear regression of RMR on age with adjustments for sex, fat mass and fat-free mass. A, 28 subjects aged 22–34 (“young”); B, 42 subjects aged 60–74 (“middle”); C, 67 nonagenarians. FI_34_ (y axis) becomes more variable (spread) in older age groups (*p*=5.8·10^−7^ for “young” vs. “middle”; *p*=0.019 for “middle” vs. nonagenarian; *p*=7.2·10^−11^ for “young” vs. nonagenarian, according to an F test to compare the variances). On the other hand, RMR (x axis) does not exhibit much change over the three age groups (*p* ≫ 0.05). Note that the red dotted line in each plot represents the correlation between resid.FI_34_ and resid.RMR. This “residual” correlation is significant only in the oldest-old group as indicated.

It is noteworthy that FI_34_ becomes more variable in older age groups, as shown previously [35], and the individual variability of FI_34_ is positively correlated with the individual variability of RMR (Figure 5). One interpretation of these results is that those among the oldest-old whose frailty markedly surpasses that of their peers have corresponding increases in RMR. This is consistent with our conclusion that elevated levels of RMR are linked to declining health in the oldest-old [46]. On the other hand, increased variability with age was not as obvious for RMR (Figure 5). According to Johannsen *et al*. [55], mean values of RMR variability declined in older age groups when compared to the 20–34 year-old group. An increase in mean RMR variability was observed from the middle-age group (60–74) to the oldest-old group (≥90), but it did not reach statistical significance. It should be noted that these were all cross-sectional findings, and longitudinal assessment may be more informative.

In contrast to RMR, TDEE remains stable; therefore, AEE would have to decline commensurately. We used the energy expenditure summary index (EESI) from the interview-based Yale Physical Activity Survey [56]. EESI summarizes the amount of energy in kilocalories spent on all the reported physical activities per week. We found EESI decreasing only in females as FI_34_ increases (Table 4). One possibility for the lack of any decrease in EESI in males is that AEE, represented by EESI here, is maintained at the expense of VO_2_ max as FI_34_ increases. VO_2_ max is the maximal oxygen uptake or maximal aerobic capacity. Whether male or female, physical ability would decline with declining health during aging, reducing physical activity. This would feed forward to further deplete physical ability, resulting in a downward spiral leading to frailty and disability.

### Genetic basis of frailty

Aging involves numerous genetic and environmental factors, each making a small contribution to the gradual development of the phenotype. Thus, no single factor would be sufficient to account for the heritable variation in aging, and longevity alone falls short of being a reliable descriptor of the actual aging process, especially in view of quality of life. This is why the idea and application of biological aging has been frequently explored in the literature [12, 57–59].

Aging accompanies progressive accumulation of age-related changes at various biological levels that decrease functional abilities and vitality [60]. Thus, genetic analysis of aging can be carried out using a single biomarker, a combination of intermediate traits, or the more inclusive FI, as long as each of these traits or measures is a significant contributor to biological aging. Incorporation of tissue-specific biomarkers, such as skeletal biomarkers, into statistical modeling and genetic analysis of underlying candidate genes has been described [59]. Skeletal muscle aging is a risk factor for geriatric diseases, and a number of factors involved in skeletal muscle metabolism, such as myokines, influence aging and life span [61]. Not surprisingly then, physical exercise stimulates autophagy [62], mitochondrial biogenesis [63], and changes DNA methylation patterns in the brain [64], along with its known effects on improvement of cognitive function [65]. It is also feasible to choose endophenotypes of healthy aging from a large number of health variables using appropriate statistical methods, such as principal component analysis [27]. No single dominant principal component could explain the bulk of the variance, but those variables that were highly correlated with the first two principal components showed high heritability.

Recent studies have shown an association between individual molecular events and frailty measures. For example, oxidative stress, as revealed by lipid and protein oxidation, is associated with phenotypic frailty based on the five standard criteria [66]. Production of interleukin-12 and interleukin-23, which play important roles in the innate immune response, is compromised in frail individuals categorized by a comprehensive geriatric assessment [67]. Analysis of the genetic factors involved in these molecular and cellular processes may help us to better understand the genetics of healthy aging. For our understanding of organismal aging, however, the use of individual biomarkers or endophenotypes is likely to yield less accurate and reliable results than does the use of comprehensive healthy aging measures [68]. Thus, it is considered to be more informative and productive to use the FI for an integrative genetic analysis than to use single or a small number of health variables.

The number of genetic studies using quantitative measures of healthy aging is small, and most of these studies are limited to linkage analysis. Reed *et al.* [69] employed a phenotype of healthy aging based on a small number of variables: reaching age of at least 70 and the absence of medical history of several major diseases. Edwards *et al.* [70] used Rowe and Kahn’s three categories of successful aging based on nine study instruments. The outcomes of these two linkage studies are different and await corroboration. Importantly, the properties of these two phenotypic measures used in linkage analyses, especially their genetic basis, are unknown. In a different approach, assuming that inflammation and muscle maintenance are associated with frailty, Ho *et al*. took a candidate gene-association approach to find SNPs and genes associated with frailty [13]. In this study, estimation of frailty was based on the five-item frailty phenotype.

We examined the genetic properties of FI_34_ [35]. First, we noted that the rates of deficit accumulation differ significantly between the offspring of long-lived parents (≥90 years old) and those of short-lived parents (<76 years old at death), indicating that FI_34_ is associated with parental longevity (Figure 2). Using 86 full sib pairs, we estimated the sib correlation coefficient to be 0.459 (95% CI=0.273–0.611) and the narrow sense heritability to be 0.39 (standard error=0.21). These results indicate that FI_34_ has a substantial genetic basis and can be used as a phenotypic measure suitable for genetic analyses of healthy aging. This has allowed us to perform a linkage analysis to identify genomic regions associated with healthy aging [71]. One such region was detected on chromosome 12. In a follow-up association analysis using a separate population, we identified three discrete healthy aging-associated sites in this genomic region coinciding with loci associated with exceptional survival [71].

## Conclusions

Various measures of biological aging have been described and used, but FI stands out for its fully quantitative nature and robustness. FIs, based on statistically valid numbers of health variables chosen to cover diverse health and body dimensions, bear common features that qualify them as reliable descriptors of healthy aging and predictors of longevity. These features of FI include its close correlation with chronological age, but its better predictive power of survival and mortality in comparison with chronological age. Using FI_34_, we found resting metabolic rate is an important physiologic factor associated with healthy aging in the oldest-old. In addition, we showed that the FI has a substantial genetic basis, which renders it suitable for genetic analysis of healthy aging and longevity. Thus, the FI can be extremely useful to study various physiologic, genetic, and epigenetic factors underlying aging and longevity.

## Figures and Tables

**Table 1:** List of 34 variables used to construct the frailty index FI_34._

No.	Name	Description	Numeric code
1	adrdz	You’ve been told that you have an adrenal disease	0, 1
2	anemia	You’ve been told that you have anemia	0, 1
3	angina	You’ve been told that you have angina	0, 1
4.	asthma	You’ve been told that you have asthma	0, 1
5	balance	Standing for 10 sec. with one foot behind the other	0, 1^a^
6	bathing	You need assistance when bathing	0, 1
7	bmi	Body mass index (BMI)	0, 0.5, 1^b^
8	bronch	You’ve been told that you have bronchitis	0, 1
9	cataracts	You’ve been told that you have cataracts	0, 1
10	chair	Number of stand-ups from chair without using arms	0, 1^c^
11	conghrtf	You’ve had congestive heart failure	0, 1
12	copd	You’ve been told that you have COPD	0, 1
13	diabetes	You’ve been told that you have diabetes	0, 1
14	dressing	You need assistance when dressing	0, 1
15	emphy	You’ve been told that you have emphysema	0, 1
16	feeding	You need assistance when eating	0, 1
17	fhoca	A first-degree relative has had cancer	0, 1
18	gds	Geriatric depression scale (GDS)[[72, 73]	0, 0,5, 1^d^
19	hattack	You’ve had a heart attack	0, 1
20	hbp	High blood pressure (based on SBP and DBP readings)	0, 0.33, 0.66, 1^e^
21	hchol	You’ve been told that you have high cholesterol	1.00
22	hhbp	You have had high blood pressure before	0, 1
23	hrtmur	You’ve been told that you have a heart murmur	0, 1
24	hrtprb	You’ve been told that you have a heart problem	0, 1
25	kidndz	You’ve been told that you have a kidney disease	0, 1
26	liverdz	You’ve been told that you have a liver disease	0, 1
27	mmse	Mini-mental state exam (MMSE)[74, 75]	0, 0.25, 0.5, 0.75, 1^f^
28	osteo	You’ve been told that you have osteoporosis	0, 1
29	seiz	You’ve had a seizure	0, 1
30	selfrated	Self-rating of health	0, 0.25, 0.5, 0.75, 1^g^
31	stroke	You’ve had a stroke	0, 1
32	thydz	You’ve been told that you have a thyroid disease	0, 1
33	tia	You’ve had a TIA	0, 1
34	urininf	You’ve been told that you have a urinary infection	0, 1

*Notes*: Reproduced with permission from [35] with modifications. COPD/copd, chronic obstructive pulmonary disease; SBP, systolic blood pressure; DBP, diastolic blood pressure; tia/TIA, transient ischemic attack. All binary variables were coded numerically: ‘0’ for the absence of the deficit and ‘1’ for its presence except where noted otherwise:

a 0 if balanced for 10 seconds, otherwise, 1;

b 0 if 18.5**≤**x**<**25, where x=weight (kg)/(height in meters)^2^, 0.5 if 25**≤**x**<** 30, otherwise, 1;

c 0 if one can stand up from chair at least once, otherwise 1;

d 0 if 0**<**x**≤**5, where x is the final score of the test, 0.5 if 6**<**x**≤**10, 1 if x**>**10;

e 0 if x**<**80 and y**<**120, where x=diastolic pressure and y=systolic pressure, 0.33 if 80**≤**x**≤**89 or 120**≤**y**≤**139, 0.66 if 90**≤**x**≤**99 or 140**≤**y**≤**159, 1 if x**≥**100 or y**≥**160. This coding is based on the categories of blood pressure levels according to the National Heart Lung and Blood Institute;

f 0 if 24**≤**x, where x is the final score of the test, 0.25 if 20**<**x**<**24, 0.5 if 18**≤**x**≤** 20, 0.75 if 10**≤**x**≤**17, and 1 if x<10;

g 0 = Excellent, 0.25 = Very good, 0.5 = Good, 0.75 = Fair, 1 = Poor.

**Table 2:** Cox regression for time to death as a function of FI_34_ or age in the Louisiana Healthy Aging Study

Variable	b	Exp (b)	*p* value	R^2^	Wald test *p*
FI_34_	2.236	9.355	0.0048	0.039	0.00482
age	0.01695	1.017	0.124	0.014	0.124

*Notes*: Reproduced with permission from [35] with modifications. The coefficient (b) and its exponentiated value, Exp (b), are for a unit increase in FI_34_. FI_34_ scores range from 0 to 1, but a FI_34_ score of 1 is practically impossible. Therefore, to better estimate the effect of the covariate, we should compute the values for a fractional increase, i.e., 0.1 rather than the whole unit [1]. In this case, e^(0.1•b)^=1.25, which means an increase in the hazard by 25% for a tenth of the unit increase in FI_34_

**Table 3:** Association of RMR and CPK with FI_34_ in “old” males and females in the Louisiana Healthy Aging Study

	“Old” male group (90–97)		“Old” female group (90–98)	

Variable	b	*p* value	b	*p* value
Age	4.03·10^−3^	0.54	−1.76·10^−3^	0.75
FM	2.26·10^−3^	0.40	4.80·10^−3^	0.014
FFM	−3.58·10^−3^	0.28	−8.17·10^−3^	0.033
TDEE	−1.42·10^−5^	0.69	−3.60·10^−5^	0.45
RMR	2.43·10^−4^	0.018	4.00·10^−4^	3.9·10^−3^
CPK	4.69·10^−4^	9.2·10^−4^	−1.29·10^−5^	0.66
IGF1	1.52·10^−5^	0.94	−9.43·10^−5^	0.60
T3	−1.83·10^−4^	0.58	−2.71·10^−4^	0.48
T4	5.03·10^−3^	0.65	1.18·10^−2^	0.14

*Notes:* Reproduced with permission from [46]. For the model FI_34_=b_0_+b_1_·age+b_2_·FM+ b_3_·FFM+b_4_·TDEE+b_5_·RMR+b_6_·CPK+b_7_·IGF1+b_8_·T3+b_9_·T4, adjusted R^2^=0.314 (*p*=0.017) for the female group and 0.349 (*p*=0.029) for the male group. Regression coefficient=b. For the “old” males, n=30, and n=37 for the “old” females. FM, fat mass; FFM, fat-free mass; TDEE, total daily energy expenditure; RMR, resting metabolic rate; CPK, creatine phosphokinase; IGF1, insulin-like growth factor 1; T3, triiodothyronine; T4, thyroxine.

**Table 4:** Association of physical-activity-related energy expenditure (EESI) with FI_34_ in female nonagenarians in the Louisiana Healthy Aging Study

Gender	b	SE(b)	*P* value	R^2^
Female	−0.917•10^−6^	3.06•10^−6^	0.0058	0.47 (*p* = 0.00052)
Male	−0.1.20•10^−6^	4.08•10^−6^	0.77	0.40 (*p* = 0.0081)

*Notes:* For the model FI_34_=b_0_+b_1_·age+b_2_·FM+b_3_·FFM+b_4_·TDEE+b_5_·RMR+b_6_·CPK+b_7_·EESI, regression coefficient=b, SE(b) is the standard error of the coefficient. For the “old” males, n=30, and n=37 for the “old” females.
